# Effectiveness of the Entomopathogenic Fungal Species *Metarhizium anisopliae* Strain NCAIM 362 Treatments against Soil Inhabiting *Melolontha melolontha* Larvae in Sweet Potato (*Ipomoea batatas* L.)

**DOI:** 10.3390/jof6030116

**Published:** 2020-07-22

**Authors:** Barna Putnoky-Csicsó, Szende Tonk, Attila Szabó, Zsuzsanna Márton, Franciska Tóthné Bogdányi, Ferenc Tóth, Éva Abod, János Bálint, Adalbert Balog

**Affiliations:** 1 Department of Horticulture, Faculty of Technical and Human Sciences, Sapientia Hungarian University of Transylvania, 400112 Cluj-Napoca, Romania; csicsobarni@gmail.com (B.P.-C.); abod.eva@ms.sapientia.ro (É.A.); 2 Department of Integrated Plant Protection, Plant Protection Institute, Faculty of Horticultural Science, Szent István University, 2100 Gödöllő, Hungary; Toth.Ferenc@mkk.szie.hu; 3 Department of Environmental Science, Faculty of Art and Sciences, Sapientia Hungarian University of Transylvania, 400193 Cluj-Napoca, Romania; tonkszende@gmail.com; 4 Department of Microbiology, ELTE Eötvös Loránd University, 1117 Budapest, Hungary; attilaszabo@ttk.elte.hu (A.S.); martonzsuzsi@caesar.elte.hu (Z.M.); 5 FKF Nonprofit Zrt., 1081 Budapest, Hungary; T.Bogdanyi.Franciska@gmail.com

**Keywords:** field production, sustainable management, pest control, soil properties, microbial community, biological activity, soil DNA analyses, α-cypermethrin

## Abstract

The effect of fungal entomopathogen *M. anisopliae* strain NCAIM 362 against *M. melolontha* larvae in sweet potato was tested under open field conditions when crop management included compost supply and soil cover (agro-foil or agro-textile). Additionally, the effect of *M. anisopliae* same strain against *M. melolontha* was compared with the effect of α-cypermethrin under greenhouse conditions. Soil microbial community using Illumina sequencing and soil biological activity were tested as possible parameter influencing *M. anisopliae* effect. According to the results, compost supply and textile cover may enhance the effectiveness of *M. anisopliae* under open field conditions, while no effect of fungal treatment was detected under greenhouse conditions. Even if soil parameters (chemical composition, bacterial, and biological activity) were identical, the effect of α-cypermethrin against *M. melolontha* larvae was significant: lower ratio of larval survival and less damaged tubers were detected after the chemical treatment. Our results suggest that *M. anisopliae* strain NCAIM 362 is not effective to control *M. melolontha* larvae, further pieces of research are needed to test other species of the *Metarhizium* genus to find an effective agent for sustainable pest control in sweet potato.

## 1. Introduction

Microbial pesticide, and especially mycoinsecticides, products based on living fungi to control arthropod pests, were given valuable research efforts in the past decades [1,2,3,4,5]. *Metarhizium* strains are soil-dwelling organisms detected extensively all over the world, regardless of climatic and soil limitations [6,7]. Members of the genus are facultative saprophytes and may either live freely within the topsoil or in the presence of a suitable arthropod host act as parasites [3,8]. The number and scope of research on *Metarhizium* species suggest that strains and isolates of *M. anisopliae* have been given the highest scientific attention within the genus, and also, they are the most widely used organisms in microbial pest control [9,10]. The first scientific recognition of *Metarhizium anisopliae* dates to Russia in 1879, when E. Metchnikoff discovered a fungus that not only covered the cadaver of a chafer, but was evidently the cause of death of the arthropod [6]. It was then named *Entomophthora anisopliae*, referring to the chafer, *Anisoplia austriaca*. Later, N.V. Sorokin repositioned this species to the genus *Metarhizium* [9,11]. When the species finds an arthropod to parasite, its conidial growth is predominantly green, giving the reason for the original name of “green muscardine” to the condition induced by the fungus [7,12].

Strains and isolates of *M. anisopliae* have long been recognized as entomopathogens, with a wide range of targeted (host) arthropods including mites, ticks, and members of the following insect orders: Diptera, Coleoptera, Hemiptera, Lepidoptera, Isoptera, Orthoptera, Thysanoptera, Homoptera, Sternorrhyncha, Heteroptera. Ongoing research of the past two decades, however, has shown that the position and effect of *M. anisopliae* is more complex. The fungus was found to colonize plants within rhizosphere, have a symbiotic relationship with plants, promote plant growth, and may act as an antagonist to plant diseases [13,14,15,16,17,18]. Commercialized products based on strains and isolates of *M. anisopliae* dominate the selection of mycoinsecticides worldwide. Formulation, application methods, targeted environment (arable or protected production), targeted crops, targeted pests, and strategies of use (inundative and non-inundative, or conservative way) are varying [4,5,10,19]. The potential of *M. anisopliae* isolates against pests of sweet potato (*Ipomoea batatas*) has been tested for more than three decades. One of the earliest virulence tests was performed in 1984, where the efficacy of three *M. anisopliae* strains were investigated in laboratory conditions against adult individuals of the sweet potato weevil (*Cylas formicarius*) [20]. A subsequent study compared 12 isolates of three fungal entomopathogens including *M. anisopliae*, also on adults of the same pest [21]. This resulted in one of the *M. anisopliae* isolates giving the lowest LD_50_ values. In another laboratory experiment, *M. anisopliae* isolates were found not only to infect and destroy coleopterans, but to have an effect on the feeding and reproduction characteristics of *Cylas puncticollis* as well [20]. It was only in 2014, when the pathogenicity of *M. anisopliae* against *C. formicarius* was evaluated not only as a standalone treatment, but in a combination with *Beauveria bassiana* [22]. The possible ways of transmitting the fungal disease in sweet potato beetle was investigated when fecundity, expressed in the number of eggs and the rate of viable eggs was significantly hindered even when the eggs themselves had no contact with the fungus. It appeared that the presence of *M. anisopliae* altered the behavior of the pest, resulting in less eggs being positioned appropriately [23].

One of the earliest accounts of testing the efficacy of the fungus in field conditions dates to 1998, when damage by the Banded Cucumber Beetle (*Diabrotica balteata*) and White grub (larvae of *Phyllophaga* spp.) were evaluated using *M. anisopliae* [24]. Although a single application before planting was found to have promising results against *D. balteata*, the effects on the other pest (i.e., Melolontha larvae) were uncertain, which may suggest that more research should be focusing on finding the conditions to enhance the efficacy of *M. anisopliae* on *M. melolontha* larvae [24].

Laboratory essays and open field experiments together suggests that, there are many abiotic and biotic factors contributing to the success and failure of using *M. anisopliae* in pest control. Among them several factors need further attention, such us soil chemical composition, soil microbiota, and biological activity [3,25]. Soil properties are governed by a complexity of factors, so in order to obtain helpful suggestions that can be used in the practice of Integrated Pest Management (IPM) or organic production, complex studies are required, with a set-up of complex models, and their viability must be trialed in realistic situations as well [26]. Altogether, more information is needed on what mechanisms endophytes establish and interact within a plant, the *M. anisopliae* on circumstances that favor the establishment of endophytism, so as to utilize its benefits [18,27,28]. Since the effect of *M. anisopliae* against *M. melolontha* larvae in sweet potato has not been a widely researched topic, we set up the present study to find answers to the following questions. (1) Can the fungal entomopathogen *M. anisopliae* strain NCAIM 362 (commercialized against coleopteran larvae) serve as an effective biological control agent against *M. melolontha* larvae in sweet potato?; (2) In sweet potato production, which soil parameters can significantly influence the efficacy of *M. anisopliae*?; (3) Is *M. anisopliae* more effective in sweet potato than the chemical insecticide?

## 2. Material and Methods

### 2.1. Experimental Set-up under Open Field and Greenhouse Conditions

**Figure 1:**
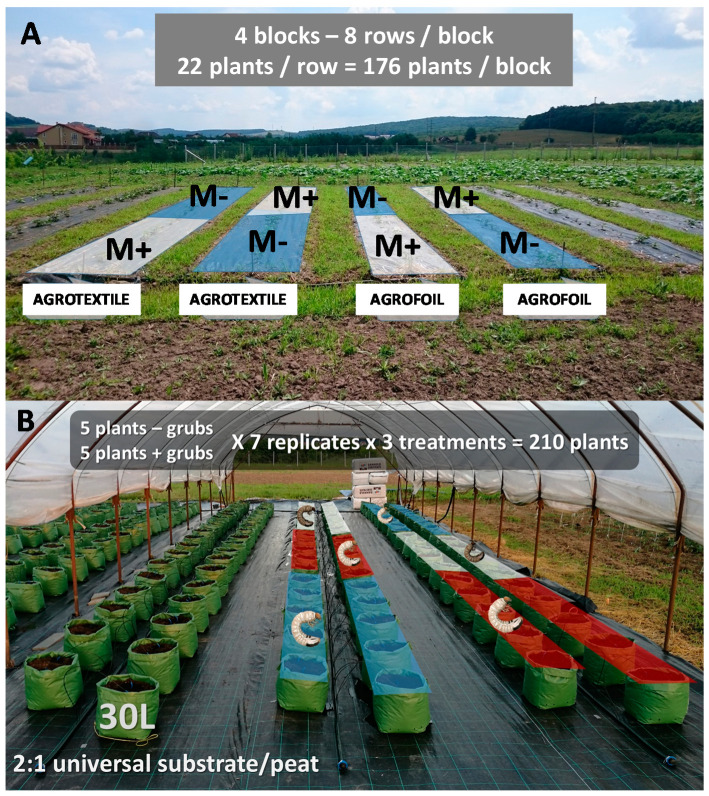
(**A,B**). Field (**A**) and greenhouse (**B**) experiment. Field experiment was replicated 4 times, having eight replicates for each cover. Greenhouse experiment was replicated seven times. Abbreviations: *M. anisopliae* present (M^+^) or absent (M^−^). Blue represents control, red represents insecticide, white represents fungal treatment.

**Figure 2:**
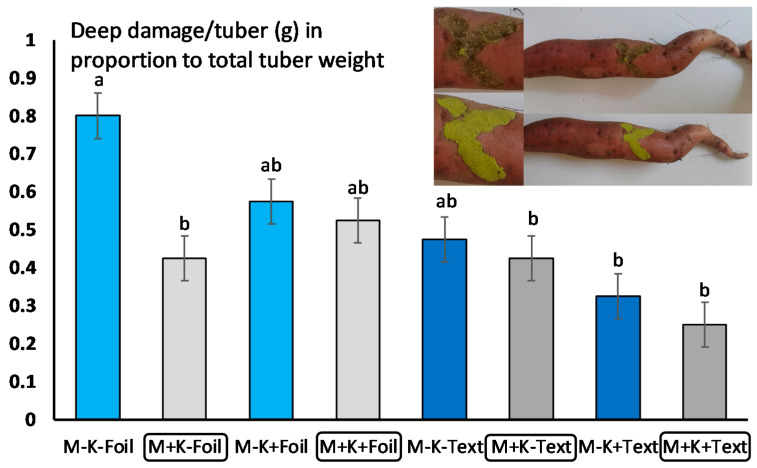
Sweet potato deep damage (defined in gram/tuber) on tubers with compost and fungal treatment and different soil cover systems. Analysis of variance (ANOVA) was used, followed by Tukey’s HSD test to compare the effect of *M. anisopliae* on tuber damages using average damage/tuber/plant/compost application/soil cover/block (*n* = 22). Grey bars represent *M. anisopliae*, blue bars represent control (no fungus). Bars represent standard errors. Upper figure presents damage assessment using gelatinized plastic. Different letters (a, b) means statistical significant differences.

Open field experiments were conducted between 2018 and 2019. Sweet potato plants Beauregard variety were obtained in 4-leaf stage from the Lajosmizse Sweet Potato Company (Lajosmizse, Hungary), and planted in eight rows/block, each row containing 22 plants. The soil was chernozem (6.5 pH). The field was chosen for our experiment because the soil inhabiting pests was dominated by *M. melolontha* larvae. This was determined before the experiment, with an average of one 3rd instar larvae/m^2^ detected. *M. melolontha* larvae infection was also influenced by the nearby (200 m distance) oak forests and orchards (100 m distance, mostly apple, pear, and plum trees at a 1.4 ha area). Since open field sweet potato production in the temperate zone usually involves the application of compost and soil cover systems (using agro-foil or textile), we followed and tested this routine. The eight rows and 22 plants within each row were also treated or not with compost and covered by agro-foil or textile (Figure 1, Figures S1 and S2). From each row, half of the plants were treated with *M. anisopliae* strain NCAIM 362 and the other half served as control (no *M. anisopliae*). There were 4 replications to each treatment, resulting in a total of eight replicates for each type. The presence of compost was marked K^+^ or K^−^; the presence of agro-foil and textile; and the presence or absence of *M. anisopliae* (M^+^ or M^−^) (Figure 1A, Figures S1 and S2). The whole system was set up at the end of May, 2018, connected to automatic irrigation system (Irritrol junior max, placed below the soil cover systems, so each plant got the same amount of water), while the *M. anisopliae* treatment in Wettable Powder (WP) formulation (as it was commercially recommended) was added on 27 June, after all plants were carefully checked. No plant pathogen symptoms or pest damages were detected on plants, and all plants were in the phenophase when the fungal entomopathogen treatment was added. This was done by preparing a 10% fungal solution (1400 g *M. anisopliae* to 12.6 L of water) transferred to all 700 plants. Treatment was added to each plant separately using a 20-mL syringe. The whole system was daily controlled until harvest. Crop harvest started on 1 October, with leaves and stems harvested first. Next, all soil covers were removed, and tubers were mechanically harvested. Each tuber from each treatment and cover system were separately collected, and tuber weights for each plant were measured and assigned to cover systems and treatments (M^+^ or M^−^). Because synthetic pesticides (i.e., α-cypermethrin) against soil inhabiting insects’ larvae are not allowed in open field sweet potato control in Europe, this treatment was only used under well controlled conditions in a greenhouse experiment. Next, the damage made by soil inhabiting insects’ larvae were evaluated using the following classification system: 0—no damage, 1—superficial damage, found only on the epidermal surface of tubers, 2—deep damage, found in deeper tissues (Figure 2). As no severe damages were detected, there was no reason to set up more levels in our classification system. The weight of missing tuber parts at level 2 damages were assessed by the following method: using gelatinized plastic with the same density as that of sweet potato tubers. Each hole was filled with this plastic. After drying (24 h), the plastic was removed and its weight (g) was measured (Figure 2). Yield was also measured at the end of the experiment by measuring every tuber under each plant. Weight results were averaged per compost use, soil cover systems, treatments and blocks. The whole experiment was replicated again in the next year, using the same cover systems, treatments, and methods.

Experimental set-up under greenhouse conditions was conducted in 2019, starting from May, parallel with the second-year field experiments. Soil properties, its chemical and microbial compositions and biological activities, were monitored under standardized and controlled conditions. The same sweet potato variety was obtained and used from the same company. For one experimental plot, there were 210 plants in three treatments (control 70 plants, fungal treatment 70 plants, and α-cypermethrin 70 plants); each divided in two sections (35 plants for each treatment) with (P^+^) and without (P^−^) *M. melolontha* larvae, all treatments replicated seven times again. Plants first were potted in 30 L plastic containers using 2:1 universal substrate/peat ensuring the same soil pH as under open field conditions. Pots were then organized in rows (Figure 1B, Figures S3 and S4). The whole system was connected to an automatic irrigation system, controlled by Irritrol junior max. Temperature inside the greenhouses were controlled and kept around 35 °C during the vegetation period. Micro and macro elements were added twice, first after potting and later, in mid-July, to each plant using automatized Dosatron^®^ systems. During the course of the whole experiment soil moisture, pH and EC were tested every three days. *M. melolontha* larvae were collected from natural environment (forest soil) from about 100 km from the experimental site and placed into the sweet potato containers when tubers were already developed, on 2 September. Two third-instar larvae were placed into each *M. melolontha*-treated container. The soil insecticide α-cypermethrin and *M. anisopliae* in a same WP formulation were added on 13 September. The insecticide was added in a concentration of 10 mL/10 L to each treated plant. The fungus was applied the same way and in the same concentration as described for the open field experiment. Tuber damage and yield weight were evaluated, as described above, too. The ratio of survived, dead, and infected *M. melolontha* larvae were counted at the end of the experiment by manually searching for larvae from containers after the plants were removed during harvest.

For soil chemical analyses, microbial assay and biological activity measurements from soil samples were collected twice: one month after planting (first week in June) and again, a month later. The same soil samples were divided and used for chemical assay, microbial analyses, and biological activity. From the soil of each treated and control plants (6 plants soil sample/treatments) 100 g soil was put into sterile pots and deposited at −70 °C until analyses. Damages on sweet potato tubers were assessed in the same way as under open field conditions.

### 2.2. Chemical Composition Assay of Sweet Potato Soil

EDX measurements were used to identify the elemental composition of the soil. Soil samples were dried in a drying cabinet at 80 °C to constant weight. Dried samples were powdered in a mortar using an electric grinder and were stored in airtight boxes. Samples were evaluated in homogenized powder form using a JEOL (Peabody, MA, USA) JSM 5510 LV scanning electron microscopy at various magnifications. The same samples were further analyzed with Scanning Jeol JEM 5510 JV and Oxford Instruments EDS Analysis System Inca 300 (UK) to determine the elemental composition of samples (*W*t%). Values are the means of five measurements from each soil samples and replicates [29,30].

### 2.3. 16S rRNA Gene Amplicon Sequencing of Soil Bacterial Community and Biological Activity Assay

The soil bacterial community analysis was performed based on amplicon sequencing of the 16S rRNA gene as in our previous work [31]. Briefly, total genomic DNA was extracted using the DNeasy PowerSoil Kit (Qiagen), a part of the 16S rRNA gene was amplified with primers containing the Bacteria-specific sequences Bakt_341F (5′-CCTACGGGNGGCWGCAG-3′; [32]) and Bakt_805NR (5′-GACTACNVGGGTATCTAATCC-3′; [33]), and DNA sequencing was performed on an Illumina MiSeq platform using MiSeq standard v2 chemistry as a service provided by the Genomics Core Facility RTSF, Michigan State University (East Lansing, MI, USA). There, Illumine-compatible, dual indexed adapters were added by PCR with primers targeting the CS1 and CS2 sites. PCR products were then batch normalized using SequalPrep DNA Normalization plates and all product recovered from the normalization plate was pooled. Subsequently, a clean-up of this pool was performed with Agencourt AMPure XP magnetic beads. Quality control and quantification was carried out using a combination of Qubit dsDNA HS (Thermo Fisher Scientific, Waltham, Massachusetts, USA), Fragment Analyzer High Sensitivity DNA (Advanced Analytical) and Kapa Illumina Library Quantification qPCR (Kapa Biosystems, Wilmington, MA, USA) assays. The pool was then loaded onto a standard MiSeq v2 flow cell Illumina. Sequencing was performed in a 2 × 250 bp paired end format using a v2, 500 cycle MiSeq reagent cartridge. Custom sequencing and index primers complementary to the CS1/CS2 oligomers were added to appropriate wells of the reagent cartridge. Base calling was done by Illumina Real Time Analysis (RTA) v1.18.54 and output of RTA was demultiplexed and converted to FastQ format with Illumina Bcl2fastq v2.19.1. Raw sequence data were submitted to NCBI under BioProject ID PRJNA632727.

For biological activity assays homogenized soil samples, sieved through a 1.6 mm sieve to remove stones and plant debris were used. For the FDA hydrolysis, 1 g of soil was measured, placed in a 500-mL conical flask, 50 mL of 100 mM potassium phosphate buffer (pH 7.6) and 0.l5 mL 12.01 μM FDA was added to start the reaction. Blank was prepared without the FDA substrate along with a control probe without soil sample. Time was monitored, and the hydrolysis took place at 37 °C for 1 h, hand-stirring every 5 min. After the hydrolysis, 2 mL of acetone were added to each probe to stop the reaction. Then the probes were centrifuged on 4000 rpm for 10 min and sieved through Whatman nr. 1 filter papers. Fluorescein concentrations were determined with spectrophotometer (PG Instruments T60 UV/VIS Spectrophotometer) on 490 nm. The obtained absorbance values were placed in the equation of calibration graph obtained by 0.03–10 μg/mL fluorescein standards, from where we obtained the FDA enzyme activities of the soil probes in μg/g soil/h. Determination was replicated three times for each sample and treatment.

### 2.4. Data Analyses

Sweet potato damage data from the field experiment were first tested for the normality of errors and homogeneity of variances. Because data were normally distributed, analysis of variance (ANOVA) was used, followed by Tukey’s HSD (Honestly Significant Difference) test to compare the effect of *M. anisopliae* on tuber damages (deep damages only) using average damage/tuber/plant/compost application/soil cover/block/treatment (*n* = 11). Data were first compared between years, then block and side effects were tested using multivariate ANOVA; MANOVA). Because no significant differences were detected between years, and no blocks and side effects detected, pooled and averaged data between years were used for further analyses. Next, crop yield (average tuber weight/plant/compost application/soil cover/fungal treatment/block (*n* = 11) were compared between control and fungal treatment using the same method (data were normally distributed).

Data from greenhouse experiment were again tested for the normality of errors and homogeneity of variances. Here, only the crop weight data was normally distributed, therefore analysis of variance (ANOVA) was used, followed by Tukey’s HSD test to compare the effect of treatments (fungal, insecticidal treatment and control) using average tuber weight (g)/plant/treatment/block (*n* = 35). Tuber damage data and *M. melolontha* larval survival and infection data did not meet the assumption of normality, therefore the nonparametric Kruskal-Wallis test was used, followed by a Mann-Whitney U test to compare damages (averaged on plants/treatments/blocks (*n* = 35)) and average survived and dead larvae (average number/plant/treatment/block (*n* = 35)). All analyses were made using R version 3.0.1 [34] and values below *p* ≤ 0.01 were considered as statistically significantly different.

Chemical composition values of the soil were compared between collection dates and between treatments using analysis of variance (ANOVA), followed by Tukey’s HSD test (data of five measurements/treatments and control).

Statistical analyses of soil bacterial communities were described in Benedek et al. [31], the differences were that the resulting sequence reads were processed using the mothur v1.41 software ([35]; based on the MiSeq standard operating procedure, downloaded on 03/04/2020) and the removal of chimeric sequences was performed using VSEARCH [36]. OTUs (operational taxonomic units) were defined at a 97% nucleotide sequence similarity level. For the statistical analysis of amplicon sequencing data, the subsampling of reads was performed to the read number of the smallest dataset (*n* = 19,791). Microbial α diversity (estimated using the Shannon-Wiener and Inverse Simpsons’s (1/D) diversity indices) and species richness values (using the Chao1 and the ACE richness metrics) were calculated using mother v1.38.1. Linear regression was used to assess the variation in total bacterial diversity indices (Shannon and Simpson) under different treatments and control, *R*^2^ values computed using PAST. Variation in bacterial community composition was also compared between genera for each treatment and control with ANOVA followed by Welch F test using mean percentages of DNA from total samples.

Data of soil biological activity was again normally distributed, thus, analysis of variance (ANOVA) was used, followed by Welch F test to compare the biological activity under different treatments and control using average data/plant/block (*n* = 6). Analyses were made using R version 3.0.1 [34].

## 3. Results

### 3.1. Field Experiment

The presence and development of *M. anisopliae* was detected both under compost and soil cover management systems. While no differences in crop weight (an average of 1600 gr/plant) were detected between treatments (Table 1), there were differences in tuber damage, and significantly higher damage (only at level 2—deep damage) was detected when the crop was covered with agro-foil, and not treated with compost and fungus. These damage figures, however, were not different from those obtained by agro-foil and compost cover, with or without fungal treatment, and textile cover without compost and *M. anisopliae* (Figure 2, Table 2). Altogether, a tendency of lower damage of Melolontha larvae was detected when sweet potato was treated with *M. anisopliae* strain NCAIM 362 and covered by agro-textile (Figure 2, Table 2).

### 3.2. Greenhouse Experiment

**Figure 3:**
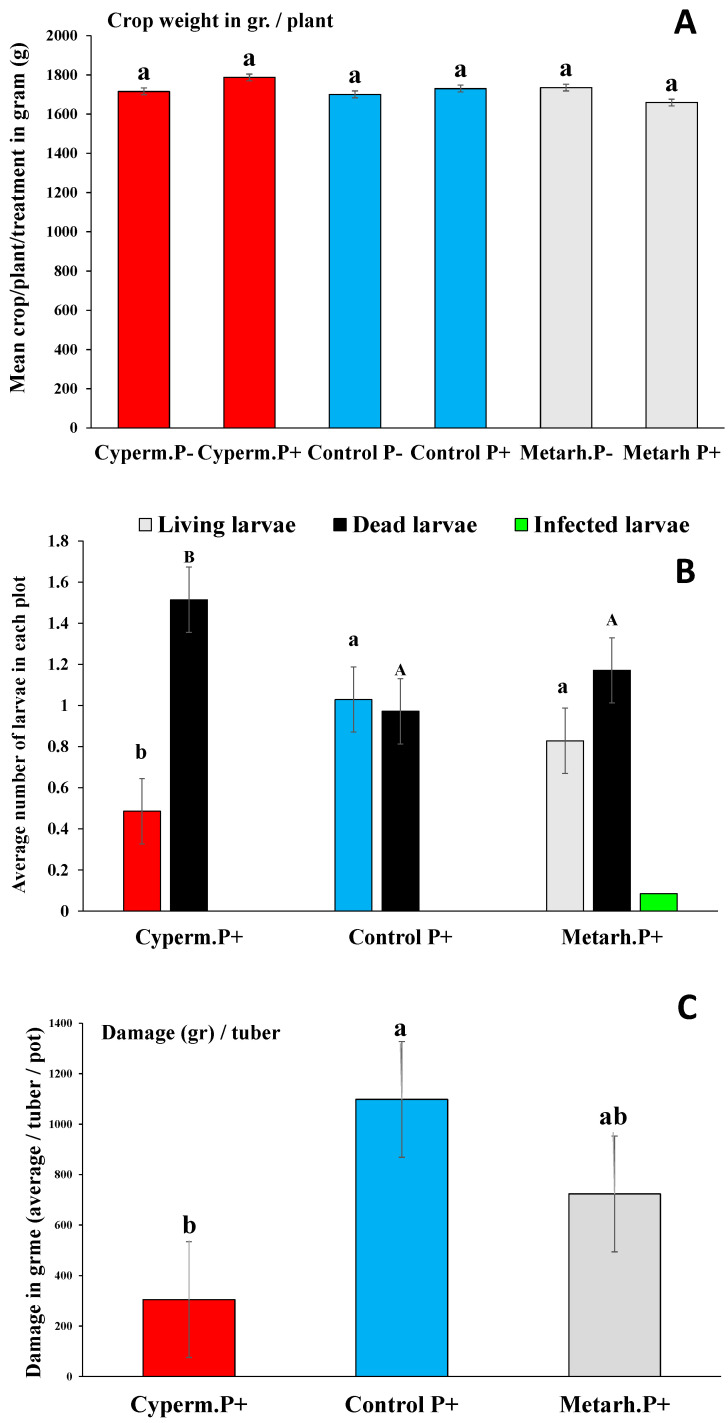
(**A**–**C**) Crop weight (g) with α-cypermethrin (red bars), control (blue bars), and fungal treatment (grey bars) (**A**); average number of alive, dead, and infected *Melolontha* larvae (**B**); and damages under different treatments and control (**C**). Crop weight data was analyzed using ANOVA, followed by Tukey’s HSD test using average tuber weight (g)/plant/treatment/block (*n* = 35). For bulb damage and *M. melolontha* larval survival data Kruskal-Wallis test was used, followed by a Mann-Whitney using average values for plants/treatments/blocks (*n* = 35)) and average values of alive, dead, and infected larvae (average number/plant/treatment/block (*n* = 35)). Bars represent standard errors. Different letters (a, b) means statistical significant differences.

Again, the presence and development of *M. anisopliae* was detected in all containers M^+^. While no differences in crop weight (an average of 1700 gr/plants) were observed (Table 3, Figure 3A), variations in *M. melolontha* larvae survival and damage were detected between treatments (Figure 3B,C). Significantly lower numbers of survived larvae were detected in plots treated with α-cypermethrin (CipermP + ControlP^+^ *U* = 3.2, *p* < 0.01; CipermP + MetarhP^+^ *U* = 3.0, *p* < 0.01) and no differences were detected between *Metarhizium* treatment and control (MetarhP + ControlP^+^ *U* = 0.67, *p* < 0.23), where generally half of the larvae died before the end of the experiment. The numbers of dead larvae were higher in plots treated with α-cypermethrin (CipermP + ControlP^+^ *U* = 4.1, *p* < 0.01; CipermP + MetarhP^+^ *U* = 3.9, *p* < 0.01) and again no differences were detected between *Metarhizium* treatment and control (MetarhP + ControlP^+^ *U* = 0.88, *p* < 0.56) (Figure 3B). Signs of fungal infection among larvae were hardly observed at all (an average of one infected larva was found in 10 *Metarhizium* treated pots) at the end of the experiment in M^+^ treatments, and this did not make statistical analysis possible (Figure 3B). The damage rate of tubers also varied between treatments. Significantly lower damage rates were detected in pots with α-cypermethrin (CipermP + ControlP^+^ *U* = 5.2, *p* < 0.01; CipermP + MetarhP^+^ *U* = 3.9, *p* < 0.01), while no differences between *Metarhizium* treatment and control were observed (MetarhP + ControlP^+^ *U* = 0.66, *p* < 0.45) (Figure 3C).

### 3.3. Chemical Composition Assay of Sweet Potato Soil

Representative elemental composition of sweet potato soil results was averaged out from five measurement each. Since a small amount of sample was used, results below 0.5 are considered as qualitative information, given that these elements only appear in trace amount. No differences in the chemical composition of sweet potato soil were detected between treatments (Table 4).

### 3.4. Microbial Community and Biological Activity in Sweet Potato Soil

**Figure 4:**
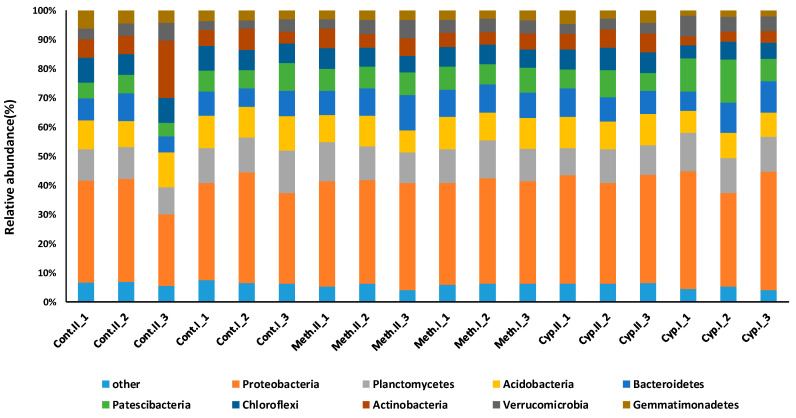
Soil bacterial community analysis performed on amplicon sequencing of the 16S rRNA gene. Total genomic DNA was extracted using the DNeasy PowerSoil Kit (Qiagen), a part of the 16S rRNA gene was amplified with primers containing the Bacteria-specific sequences Bakt_341F (5′-CCTACGGGNGGCWGCAG-3′; Herlemann et al., 2011) and Bakt_805NR (5′-GACTACNVGGGTATCTAATCC-3′). DNA sequencing was performed on an Illumina MiSeq platform using MiSeq standard v2 chemistry as a service provided by the Genomics Core Facility RTSF, Michigan State University, USA. Cont.II represents control P^−^, Cont.I represents control P^+^, Meth.II represents Metarhizium P^−^, Meth.I represents Metarhizium P+, Cyp.II represents insecticide P^−^ treatments, Cyp.I represents insecticide P+. Only data of 3 sample/treatment are presented.

**Figure 5:**
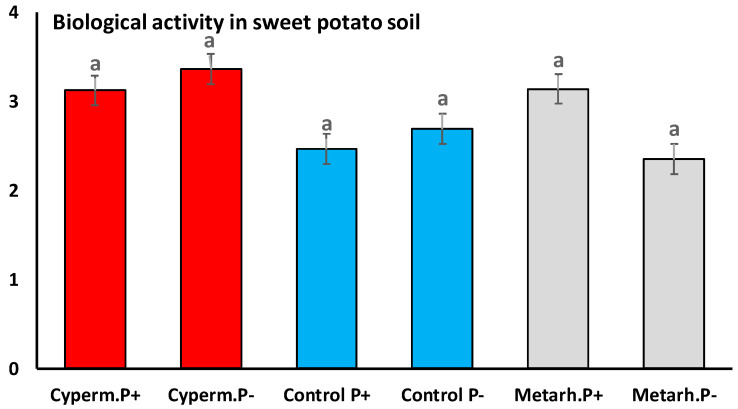
Soil biological activity with insecticide, *M. anisopliae* treatment, and control. The concentration of fluorescein was determined with spectrophotometer (*PG Instruments T60 UV/VIS Spectrophotometer*) on 490 nm. The FDA enzyme activities of the soil probes performed in μg/g soil/h. Same letters indicate no significant differences (Tukey HSD test).

A total of 697,221 high-quality bacterial 16S rRNA gene sequences were obtained from the samples (38,734 ± 9 336 reads per sample). Good’s coverage values were higher than 0.94 in all cases, which indicated that sequencing depth was sufficient to recover all major bacterial taxa (Figures S5–S7, and Table S1). The average length of sequences was ~450 nt, which allowed genus-level taxon identification. No significant differences between bacterial community were detected when the soil was treated with insecticide or the fungus, and control with and without *M. melolontha* larvae (Welch *F* test *F* = 0.0006, *df* = 22.37, *p* < 0.9) (Figure 4). Also, no significant differences in soil biological activity were detected when treatments and control were compared Welch *F* test *F* = 0.03, *df* = 6, *p* < 0.76 (Figure 5).

## 4. Discussion

The number of studies investigating the effect of *M. anisopliae* on *M. melolontha* larvae is low, although Horaczek and Vierstein (2004) mention that *Beauveria bassiana* and *M. anisopliae* have significance in controlling soil-inhabiting pests of various genera, including *Melolontha* [37]. A still earlier study reported that while a single application of *M. anisopliae* before planting was found to have promising results against *D. balteata*, the effects on *Melolontha* larvae, however, was not significant [24]. Our study showed that under open field conditions, with and without compost and different soil cover (agro-foil or textile), damage by *Melolontha* larvae was lower when sweet potato was treated with *M. anisopliae* strain NCAIM 362 in WP formulation and covered by textile (Figure 2, Table 2). A similar effect was detected by other authors on the larvae of *Polyphylla fullo* (Coleoptera: Scarabaeidae) when the highest mortality rates of young and older larvae caused by a *M. anisopliae* product were 74.1 and 67.6% for the granular formulation [38]. By comparing the effect of *M. anisopliae* with soil insecticide α-cypermethrin, a significantly lower number of survived larvae were detected with α-cypermethrin, and no differences were detected between *Metarhizium* treatment and control (Figure 3B). Even when *Metarhizium* concentrations were tripled (1400 g *M. anisopliae* to 12.6 l. of water) compared to the commercially suggested dosage, only 50% of the larvae died on average by the end of the experiment in M^+^ treatments. Also, the number of dead larvae were higher with α-cypermethrin, and again no differences were detected between *Metarhizium* and control (Figure 3B). Altogether, this suggests that *Metarhizium* strain NCAIM 362 in WP formulation (recommended and commercialized against coleopteran larvae) is less effective than α-cypermethrin against *Melolontha* larvae, making its future application in sweet potato control uncertain. This can also be supported by the fact that only a low amount of *Melolontha* larvae were observed to have signs of fungal infection (an average of one infected larvae in 10 *Metarhizium* treated pots) at the end of the experiment in M^+^ treated pots (Figure 3B). The damage rate of sweet potato tubers had a strong connection with larval survived rate. Significantly lower damages were observed with α-cypermethrin, and no differences were detected between *Metarhizium* and control detected. Recent studies also reported the effect of *Beauveria brongniartii* (three isolates) and *M. anisopliae* (three isolates) on *M. melolontha, Amphimallon solstitiale,* and *Anoxia villosa* under laboratory conditions. The highest mortality rates were caused by *B. brongniartii* isolates (100%) on *M. melolontha* larvae, and 60% mortality on *A. villosa*. In comparison, *A. villosa* was found the most susceptible to *M. anisopliae* (35.5% mortality rate), and the fungus had little to no significant effect on *A. solstitiale* or on *M. melolontha* [39]. In our experiment, no differences were detected in the chemical composition of the soil, in the microbial community, and biological activity of the soil either between treatments and control. The fungal effects on *Melolontha* larvae were completed under very similar conditions, yet no effects on larval mortality and thereby on tuber damage were detected, suggesting that *M. anisopliae* strain NCAIM 362 cannot effectively control *M. melolontha* in sweet potato. This can be explained in different ways. The apparent resistance of *Melolontha* larvae against *M. anisopliae* is hard to be explained without further research. One possible reason can be the fact that a long evolutionary interrelation exists between this soil inhabiting larva and the fungus, meaning that a genetic resistance may have evolved. The effects of formulation may also result in different characteristics of the fungus including conidial growth, viability, potential to cause mortality to target organisms, persistence, and resistance to certain environmental factors. One may also notice that while the impact of formulation on fungal performance was intensively researched in the 1990s, the number of research pieces conducted in this area has been lower ever since. One inevitable challenge formulation faces when trying to enhance the efficiency of the fungal entomopathogen is the presence of ultra-violet light among unprotected field conditions that has a significant negative effect on *M. flavoviride* germination [40]. The frequency of bacterial genera were similar for both treatment and control. Most bacterial taxons identified as dominant (Proteobacteria, Planctomycetes, Acidobacteria, Bacteroidetes, Patescibacteria, Chloroflexi), are known to have a significant role in litter biodegradation and mineralization processes [31]. While no high variation in bacterial community were detected between control and treatment, means that no soil inhabiting microorganisms with inhibitory effect on *M. flavoviride* were detected. These further demonstrate that, *M. flavoviride’s* effects were tested under ideal conditions.

These environmental effects on *M. anisopliae* need further, more detailed tests. When looking for a successful pest control species, other species of the Metarhizium genus may have more potential against Melolontha larvae. In 2015, a genetic characterization studies performed on fungal isolates obtained from fungus-infected larvae of the Coleopteran *Amphimallon solstitiale* collected from roots of various plants in north eastern Turkey revealed that the hosts were infected by *M. flavoviride* [41]. Finally, a series of experiments, including this present one indicate that the effect of *M. anisopliae* on *Melolontha* larvae is non-significant, therefore the effect of other *Metarhizium* species (e.g., *M. flavoviride*) as an effective control agent in sustainable management needs to be investigated.

## 5. Conclusions

According to the results, the effect of fungal entomopathogen *M. anisopliae* strain NCAIM 362 in WP formulation against *M. melolontha* larvae in sweet potato is not an effective biological control method. Even if the soil parameters are identical, the effect of α-cypermethrin against *Melolontha* larvae is more significant, and less survived larvae and damaged tubers can be detected after the insecticidal treatment. Under open field conditions, some soil management methods such as compost supply and textile cover may enhance the effect of *M. anisopliae*, but further research is needed to test other species of the *Metarhizium* genus to find if they are an effective agent in sweet potato sustainable pest control.

## Figures and Tables

**Table 1:** Statistical analyses of tuber weight in the open field experiment (F values below and *p* values above line), data were compared using average tuber weight/plant/compost application/soil cover/block (*n* = 22).

Treatments	M-K-Foil	M + K-Foil	M-K + Foil	M + K + Foil	M-K-Text	M + K-Text	M-K + Text	M + K + Text
M-K-Foil	-	0.984	0.992	0.999	1	1	1	0.995
M + K-Foil	1.27	-	1	0.999	0.999	0.963	0.906	1
M-K + Foil	1.132	0.137	-	0.999	0.999	0.978	0.936	1
M + K + Foil	0.538	0.731	0.593	-	1	0.999	0.994	1
M-K-Text	0.513	0.756	0.618	0.024	-	0.999	0.995	0.999
M + K-Text	0.221	1.491	1.354	0.76	0.735	-	1	0.984
M-K + Text	0.520	1.790	1.653	1.059	1.034	0.298	-	0.950
M + K + Text	1.058	0.212	0.074	0.519	0.544	1.279	1.578	-

**Table 2:** Statistical analyses of tuber damages in the open field experiment (*F* values below and *p* values above line), the effect of *M. anisopliae* on deep tuber damages (level 2) were compared using average damage/tuber/plant/compost application/soil cover/block (*n* = 22). Bold numbers represent statistically significant *p* values.

Treatments	M-K-Foil	M + K-Foil	M-K + Foil	M + K + Foil	M-K-Text	M + K-Text	M-K + Text	M + K + Text
M-K-Foil	-	**0.013**	0.153	0.113	0.060	**0.010**	**0.004**	**0.0008**
M + K-Foil	4.453	-	0.483	0.422	0.593	0.896	0.661	0.333
M-K + Foil	0.134	0.101	-	0.986	0.815	0.420	0.248	0.099
M + K + Foil	0.121	0.211	0.431	-	0.774	0.359	0.190	0.063
M-K-Text	0.145	0.322	0.561	0.981	-	0.511	0.302	0.113
M + K-Text	5.061	0.111	0.431	0.789	0.891	-	0.778	0.426
M-K + Text	6.275	0.321	0.671	0.671	0.791	0.991	-	0.580
M + K + Text	8.334	0.451	0.451	0.961	0.954	0.781	0.871	-

**Table 3:** Statistical analyses of tuber weight in the greenhouse. * Significance: same letters indicate no significant differences (Tukey HSD test).

Treatments	Tuber Weight/Plant (g)	*
Cypermethrin P^−^	1716.19	a
Cypermethrin P^+^	1787.46	a
Control P^−^	1700.85	a
Control P^+^	1730.68	a
Metarhizium P^−^	1735.36	a
Metarhizium P^+^	1659.32	a

**Table 4:** Representative elemental composition of sweet potato soil. Results were obtained by calculating the average of five measurements. * Significance: same letters indicate no significant differences (Tukey HSD test).

Elements	ControlP^+^	ControlP^−^	Metarh.P^+^	Metarh.P^−^	Cyperm.P^+^	Cyperm.P^−^	*
C	47.379	41.403	47.043	44.720	51.307	47.773	a
Na	0.122	0.108	0.109	0.079	0.191	0.111	a
Mg	0.328	0.334	0.330	0.414	0.354	0.265	a
Al	1.715	1.668	1.606	2.133	1.261	1.501	a
Si	4.648	7.114	4.184	5.816	3.131	6.456	a
P	0.131	0.220	0.132	0.158	0.133	0.104	a
S	0.251	0.150	0.210	0.348	0.220	0.148	a
Cl	0.022	0.010	0.016	0.011	0.019	0.019	a
K	0.623	0.395	0.364	0.540	0.587	0.242	a
Ca	3.255	4.570	3.706	2.198	1.968	1.908	a
Ti	0.087	0.100	0.077	0.140	0.070	0.048	a
Mn	0.040	0.068	0.041	0.072	0.010	0.020	a
Fe	1.362	2.337	1.122	1.294	0.876	0.722	a
Cu	0.216	0.202	0.253	0.348	0.246	0.310	a
Zn	0.186	0.152	0.222	0.272	0.188	0.253	a
Mo	0.038	0.029	0.030	0.098	0.092	0.086	a

## Supplementary Materials

The following are available online at https://www.mdpi.com/2309-608X/6/3/116/s1, Figure S1. The scheme of the field experiment, Figure S2. Plots of the field experiment with or without foil cover, Figure S3. Experimental greenhouse at the beginning of the season, Figure S4. Experimental greenhouse in the middle of the season, Figure S5. Genus-level of the bacterial composition of sweet potato soil, Figure S6. NMDS ordination of soil samples based on the Bray-Curtis distance of the bacterial OTUs, Figure S7. Rarefaction curves of OTUs based on 16S rRNA gene amplicon sequencing data, Table S1. Bacterial species numbers (sobs, ACE and Chao-1) and diversity indices (inverse Simpson and Shannon-Weaver) calculated from 16S rRNA gene amplicon sequencing data. KI—represents control P+, KII—represents control P-, MI – represents Metarhizium P+, MII—represents Metarhizium P-, VI- represents insecticide P+, VII- represents insecticide P- treatment.

## Abbreviations

Compost present	K^+^
No compost	K^−^
*M. anisopliae* present	M^+^
No *M. anisopliae*	M^−^
*M. melolontha* grubs present	P^+^
No *M. melolontha*	P^−^
