# Supplementary material for: Effectiveness of the Entomopathogenic Fungal Species Metarhizium anisopliae Strain NCAIM 362 Treatments against Soil Inhabiting Melolontha melolontha Larvae in Sweet Potato (Ipomoea batatas L.)
Source: J Fungi (Basel). 2020 Jul 22;6(3):116. doi: 10.3390/jof6030116 (PMC7560189; doi:10.3390/jof6030116)
Supplement: Supplementary file 1 [file jof-06-00116-s001.pdf]

Supplementary online material

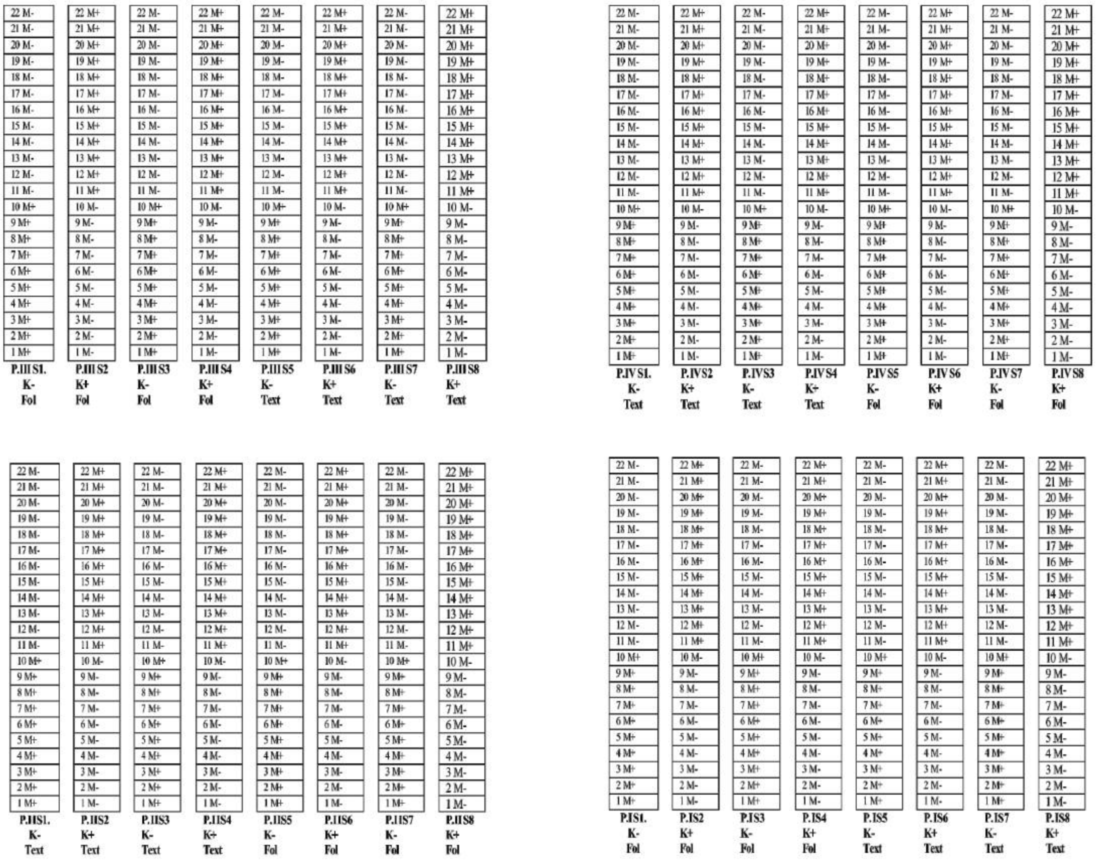

Figure S1. The scheme of the field experiment.

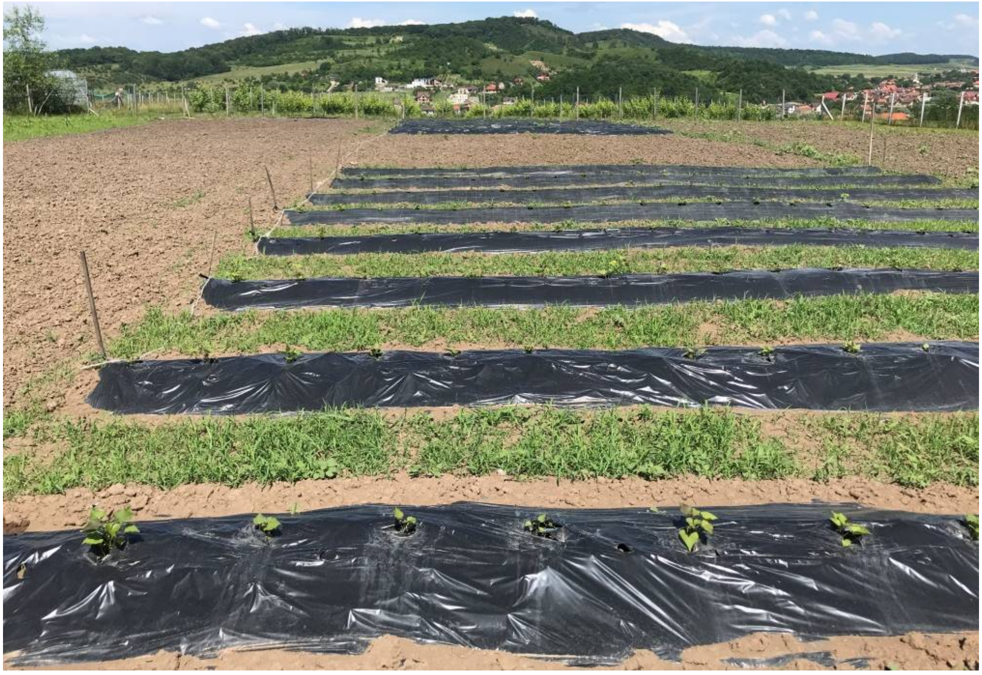

Figure S2. Plots of the field experiment with or without foil cover.

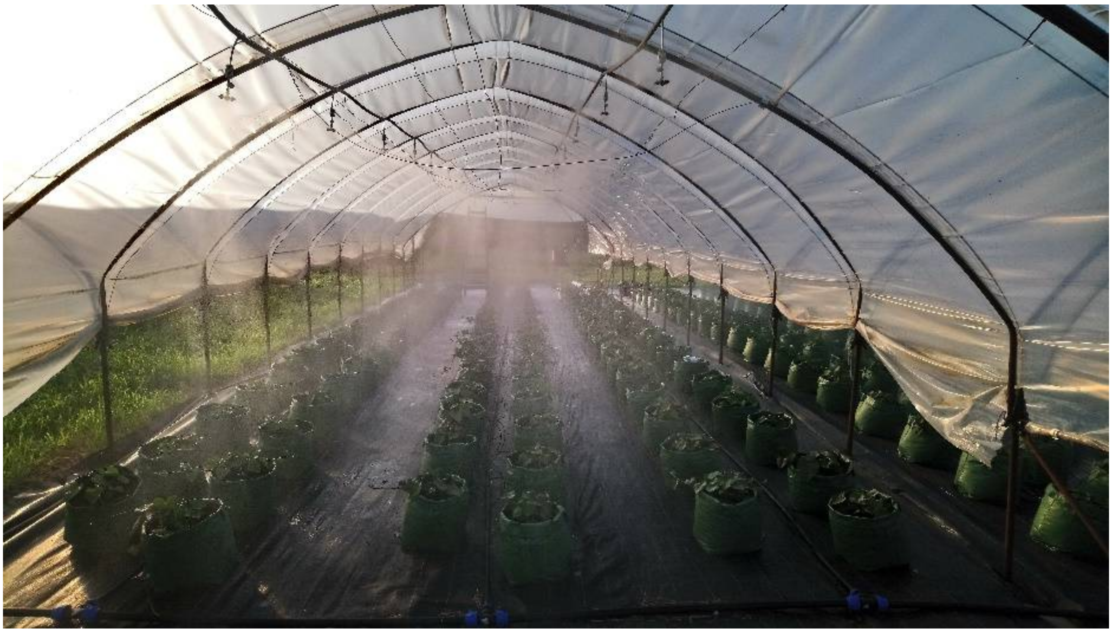

Figure S3. Experimental greenhouse at the beginning of the season.

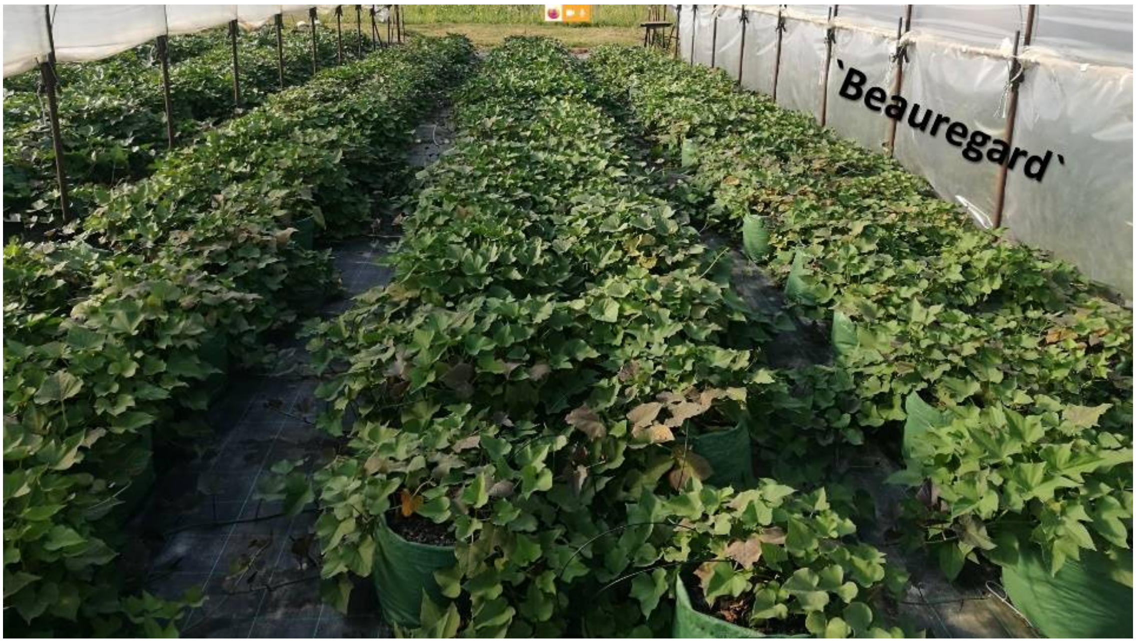

Figure S4. Experimental greenhouse in the middle of the season.

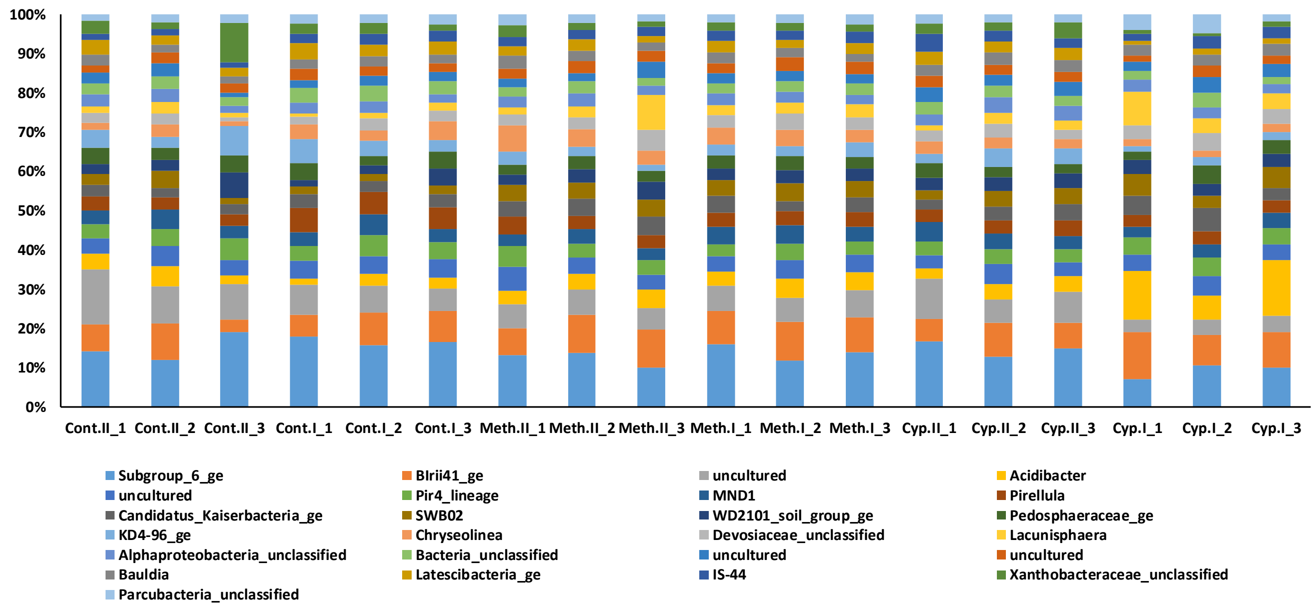

Figure S5. Genus-level of the bacterial composition of sweet potato soil. Only the top 24 most abundant genera are shown. Total genomic DNA was extracted using the DNeasy PowerSoil Kit (Qiagen), a part of the 16S rRNA gene was amplified with primers containing the Bacteria-specific sequences Bakt_341F (5’-CCT ACG GGN GGC WGC AG-3′; Herlemann et al., 2011) and Bakt_805NR (5’-GAC TAC NVG GGT ATC TAA TCC-3′). DNA sequencing was performed on an Illumina MiSeq platform using MiSeq standard v2 chemistry as a service provided by the Genomics Core Facility RTSF, Michigan State University, USA. Cont.II–represents control P-, Cont.I–represents control P+, Meth.II – represents Metarhizium P-, Meth.I – represents Metarhizium P+, Cyp.II- represents insecticide P- treatments, Cyp.I- represents insecticide P+.

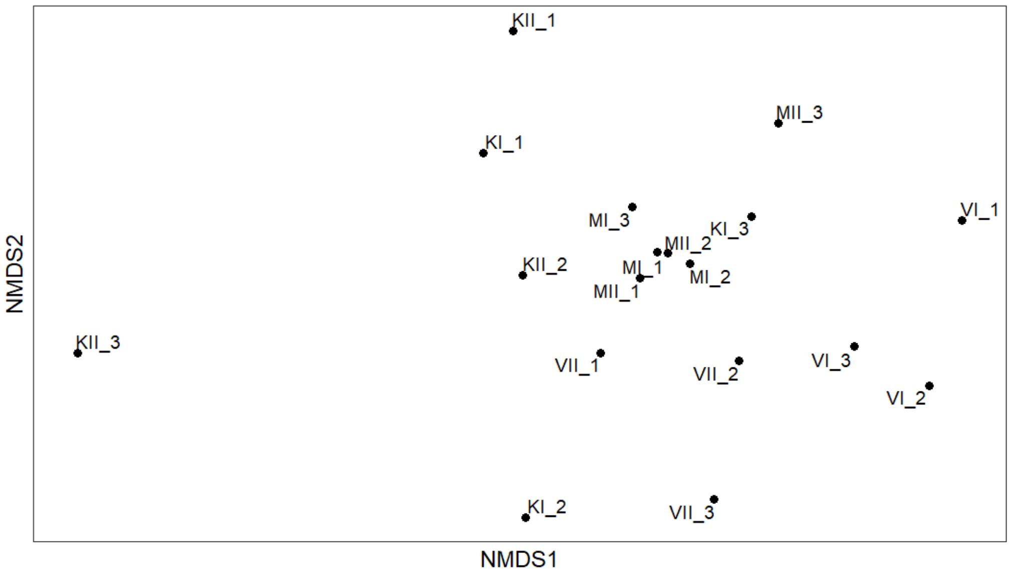

Figure S6. NMDS ordination of soil samples based on the Bray-Curtis distance of the bacterial OTUs. KI–represents control P+, KII–represents control P-, MI – represents *Metarhizium* P+, MII – represents *Metarhizium* P-, VI- represents insecticide P+, VII- represents insecticide P- treatment.

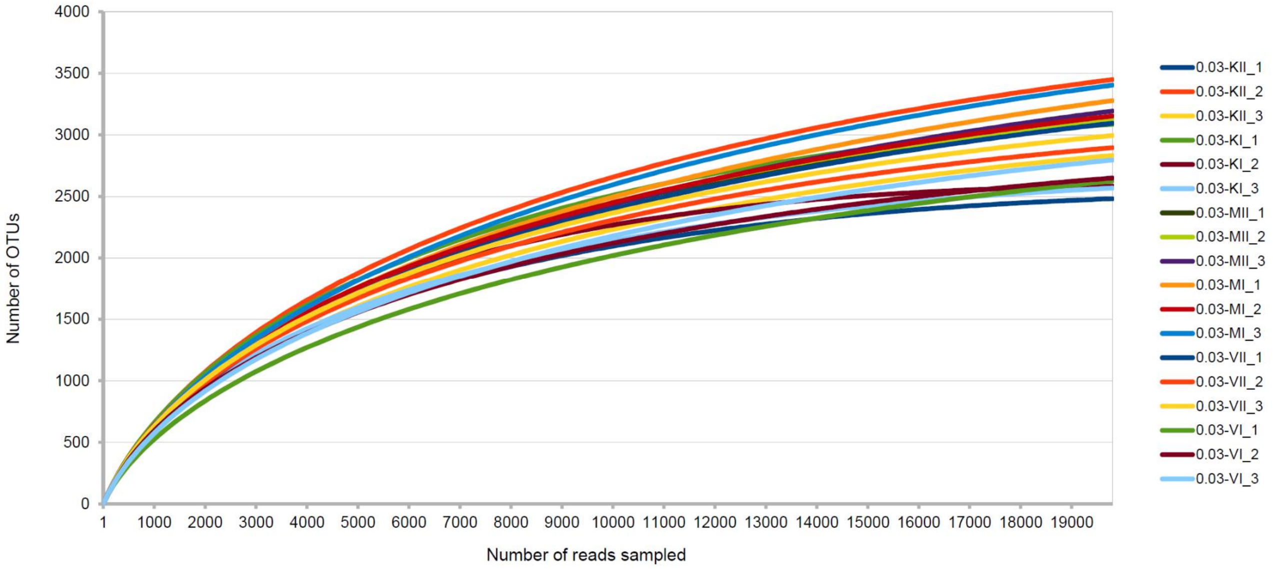

Figure S7. Rarefaction curves of OTUs based on 16S rRNA gene amplicon sequencing data. KI– represents control P+, KII–represents control P-, MI – represents *Metarhizium* P+, MII – represents *Metarhizium* P-, VI- represents insecticide P+, VII- represents insecticide P- treatment.

Table S1. Bacterial species numbers (sobs, ACE and Chao-1) and diversity indices (inverse Simpson and Shannon-Weaver) calculated from 16S rRNA gene amplicon sequencing data. KI–represents control P+, KII–represents control P-, MI – represents *Metarhizium* P+, MII – represents *Metarhizium* P-, VI- represents insecticide P+, VII- represents insecticide P- treatment.

**Sample**	**Number of sequences**	**Coverage**	**sobs**	**ACE**	**Chao-1**	**inv. Simpson**	**Shannon-Weaver**
**KII_1**	23 548	0.985	2476	2632	2545	489	6.96
**KII_2**	43 528	0.948	3423	4335	4080	524	7.24
**KII_3**	37 745	0.962	2835	3435	3251	364	6.91
**KI_1**	29 118	0.969	3092	3491	3324	700	7.25
**KI_2**	19 791	0.994	2585	2624	2593	595	7.13
**KI_3**	26 241	0.979	2574	2810	2702	484	6.99
**MII_1**	39 091	0.956	3123	3836	3622	555	7.14
**MII_2**	39 288	0.956	3132	3851	3638	528	7.16
**MII_3**	46 541	0.949	3173	4105	3865	289	6.94
**MI_1**	54 173	0.944	3283	4355	4129	494	7.13
**MI_2**	47 644	0.951	3195	4063	3878	486	7.15
**MI_3**	48 376	0.943	3407	4506	4227	482	7.16
**VII_1**	44 907	0.952	3078	3952	3730	564	7.11
**VII_2**	36 406	0.964	2877	3408	3241	503	7.05
**VII_3**	39 339	0.960	2990	3635	3455	570	7.13
**VI_1**	44 735	0.958	2645	3433	3219	150	6.54
**VI_2**	32 558	0.969	2659	3107	2947	375	6.93
**VI_3**	44 192	0.958	2809	3535	3347	193	6.80
